# MicroRNA-145 suppresses cell proliferation, invasion and migration in pancreatic cancer cells by targeting NEDD9

**DOI:** 10.3892/mmr.2015.3294

**Published:** 2015-02-03

**Authors:** TONG HAN, XIAO-PING YI, BO LIU, MU-JING KE, YI-XIONG LI

**Affiliations:** 1 Departments of General Surgery, Central South University, Changsha, Hunan 410008, P.R. China; 2 Departments of Radiology, Xiangya Hospital, Central South University, Changsha, Hunan 410008, P.R. China; 3 State Key Laboratory of Medical Genetics, Central South University, Changsha, Hunan 410008, P.R. China

**Keywords:** pancreatic cancer, microRNA-145, neural precursor cell expressed, developmentally downregulated 9, invasion, migration

## Abstract

MicroRNAs (miRNAs) represent a class of small non-coding RNAs regulating gene expression by inducing the degradation of RNA or interfering with translation. Aberrant miRNA expression has been described in several types of cancer in humans. In the present study, it was demonstrated that miR-145 is downregulated in pancreatic cancer tissues and the Panc-1 cell line. Restoration of miR-145 inhibited cell proliferation, invasion and migration in Panc-1 cells. Neural precursor cell expressed, developmentally down-regulated 9 (NEDD9) has been identified as a novel potential miR-145 target using bioinformatics. Using luciferase reporter constructs, it was observed that the NEDD9 3′-untranslated region is the location of the direct binding site for miR-145. Additionally, it was identified that miR-145 is inversely correlated with NEDD9 expression in pancreatic cancer tissues and that restoration of miR-145 in Panc-1 cells reduced NEDD9 mRNA and protein expression accompanied by inhibition of cell proliferation, invasion and migration. In conclusion, these findings indicate that miR-145 may be an effective target for pancreatic cancer therapy.

## Introduction

Pancreatic cancer (PC) is characterized by rapid invasion, early metastasis and resistance to current standard therapies (1). Understanding of the molecular mechanisms in PC is urgently required in order to identify novel treatment strategies. MicroRNAs (miRNAs, miRs) are endogenous short non-coding RNA molecules consisting of 17–25 nucleotides, which have been frequently observed to be dysregulated in diverse types of human cancer (2–4). The functions of miRNAs as either oncogenes or tumor suppressors has generated interest as to their possible use as novel targets or tools for anticancer therapies (5,6).

miR-145 was identified as a tumor-suppressive miRNA, which is downregulated in several types of cancer, including prostate, bladder, breast, lung and ovarian cancer (7–12). Microarray studies have revealed that miR-145 is also down-regulated in PC (13,14). However, the biological function of miR-145 in PC remains to be fully elucidated. In the present study, it was identified that miR-145 is downregulated in PC tissues compared with matched normal adjacent pancreatic tissues. Notably, it was identified that neural precursor cell expressed, developmentally downregulated 9 (NEDD9, also termed HEF1 or Cas-L), a non-catalytic scaffolding protein implicated in the invasive ability of several types of cancer (15,16), is a novel target of miR-145 in PC. Restoration of miR-145 suppresses Panc-1 cell proliferation, invasion and migration through reducing levels of NEDD9.

## Materials and methods

### Cell lines and human tissue samples

The Panc-1 human pancreatic ductal adenocarcinoma (PDAC) cell line was obtained from the American Type Culture Collection (Manassas, VA, USA). All cells were cultured in Dulbecco’s modified Eagle’s medium (Invitrogen Life Technologies, Carlsbad, CA, USA) supplemented with 10% fetal bovine serum (Invitrogen Life Technologies) at 37°C in a humidified atmosphere containing 5% CO_2_. A total of 20 PDAC tissues and paired normal adjacent pancreatic tissues were obtained from the patients who were confirmed to have PDAC by pathological analysis and underwent the Whipple procedure at the Department of General Surgery, Xiangya Hospital, Central South University (Changsha, China). Specimens were flash-frozen in liquid nitrogen immediately and stored at −80°C for future use. The protocol for the study was approved by the Xiangya Hospital Clinical Research Ethics Committee.

### miRNA transfection

miR-145 mimics and negative control miRs (miR-NC) were synthesized by Yingrun Biotechnology Inc. (Changsha, China). A total of 1×10^4^ Panc-1 cells/well were seeded into six-well plates and then cells were transfected in a solution with 75 nM miR-145 mimics or miR-NC (Yingrun Biotechnology Inc.) using Lipofectamine 2000 (Invitrogen Life Technologies) according to the manufacturer’s instructions. Total RNA and protein were extracted at 48 h post-transfection and used for reverse transcription-quantitative polymerase chain reaction (RT-qPCR) and western blot analysis.

### RNA isolation and RT-qPCR

Total RNAs were isolated using TRIzol reagent (Invitrogen Life Technologies) according to the manufacturer’s instructions. The miR-145 levels were assayed using TaqMan MicroRNA assays (Applied Biosystems, Carlsbad, CA, USA). The u6 small nuclear B noncoding RNA (Applied Biosystems) level was used as an internal normalization control. For NEDD9 mRNA analysis the primer sequences were as follows: Forward: 5′-GAGCTGGATGGATGACTACGA-3′ and reverse: 5′-AGCTCTTTCTGTTGCCTCTCA-3′. Total RNA was reverse-transcribed with the SuperScript III first-strand synthesis system for RT-PCR (Invitrogen Life Technologies) and amplified with 2X SYBR Green real-time PCR master mix (Toyobo, Osaka, Japan). PCR was conducted using an ABI 7500 Real-Time PCR System (Applied Biosystems) and the results were normalized. Relative fold changes were calculated using the 2^∆∆Ct^ method and standard curves were produced. β-actin was used as an internal normalization control.

### Western blot analysis

At 48 h after transfection, Panc-1 cells were lysed with radioimmunoprecipitation assay lysis buffer and proteins were harvested. Proteins were resolved on an SDS denatured polyacrylamide gel and then transferred onto a nitrocellulose membrane (Millipore Corp., Billerica, MA, USA). A mouse anti-human anti-NEDD9 polyclonal antibody (1:1,000; 4044S; Cell Signaling Technology Inc., Beverly, MA, USA) and a mouse anti-human anti-β-actin polyclonal antibody (1:4,000; sc-130301; Santa Cruz Biotechnology, Inc., Santa Cruz, CA, USA) were incubated with the blot overnight at 4°C. Membranes were washed and incubated with goat anti-mouse secondary antibodies (1:10,000) and were visual-ized by enhanced chemiluminescence (Millipore, Billerica, MA, USA) according to the manufacturer’s instructions.

### Prediction of miRNA targets

In order to investigate the predicted target genes, the TargetScan program (http://www.targetscan.org/), the miRanda program (http://microrna.sanger.ac.uk), the miRWalk program (http://www.umm.uni-heidelberg.de/apps/zmf/mirwalk/) and the PicTar4 program (http://pictar.bio.nyu.edu) were used. The TargetScan program (http://www.targetscan.org/) was used to predict the seed region.

### Plasmid construction and the dual-luciferase assay

For the validation of NEDD9 as a direct target of miR-145, an miRNA target luciferase reporter assay was performed using a target reporter plasmid containing wild-type (WT) NEDD9 3′-untranslated region (UTR) and mutant NEDD9 3′UTR. Co-transfection experiments were performed in 96-well plates. A total of 1×10^4^ HEK-293 cells (American Type Culture Collection, Manassas, VA, USA) were seeded per well in 200 *μ*l medium. A total of 100 ng WT or mutant (MT) reporter constructs were co-transfected with Lipofectamine 2000 transfection reagent into the PC cells with 50 nM miR-145 mimics or miR-NC according to the manufacturer’s instructions. After 48 h, the luciferase activity was measured with the dual luciferase reporter assay system (Promega Corporation, Madison, WI, USA). The relative luciferase activity was normalized to that of firefly luciferase.

### Cell proliferation

Cell proliferation was determined using an MTT assay. Briefly, the Panc-1 cells were seeded in 96-well culture plates at a density of 1×10^4^ cells per well. After 24 h of culturing, the cells were transfected with 75 nM miR-145 mimics or miR-NC using Lipofectamine 2000. The cells were then cultured in the medium and proliferation rates at 1, 2, 3, 4 and 5 days after transfection were assessed by a colorimetric assay using 5 mg/ml MTT solution at 490 nm. All the experiments were performed three times with five replicates.

### Cell migration and invasion analysis

Panc-1 cells were transfected with 75 nM miR-145 mimics or miR-145 NC for 48 h, trypsinized and plated for migration and invasion assays. For the migration assay, 5×10^4^ cells were plated in the top chamber of monocoated polyethylene teraphthalate membrane (6-well insert, 8 *μ*m pore size; BD Biosciences, Bedford, MA, USA). For the invasion assay, 2×10^5^ cells were plated in the top chamber of the transwell with a Matrigel-coated polycarbonate membrane (24 wells insert, 8 *μ*m pore size; BD Biosciences). Respective medium with 10% fetal bovine serum (Invitrogen Life Technologies) was added to the lower chamber as a chemoattractant. After 24 h of incubation, cells remaining on the upper surface of the insert membrane were removed using a cotton swab. Cells, which had migrated or invaded through the membrane/Matrigel to the bottom of the insert were stained with 0.1% crystal violet for 30 min at 37°C and washed with phosphate-buffered saline. Finally, migrated or invaded cells were counted under a microscope (IX71; Olympus Corp., Tokyo, Japan) in five random visual fields and the relative number was calculated.

### Statistical analysis

Each experiment was conducted at least three times. All values are expressed as the mean ± standard deviation. Differences between experimental groups and controls were assessed via Student’s t-test using Excel software (Microsoft Inc., Redmond, WA, USA). P<0.05 was considered to indicate a statistically significant difference.

## Results

### miR-145 expression is downregulated in PC

**Figure 1:**
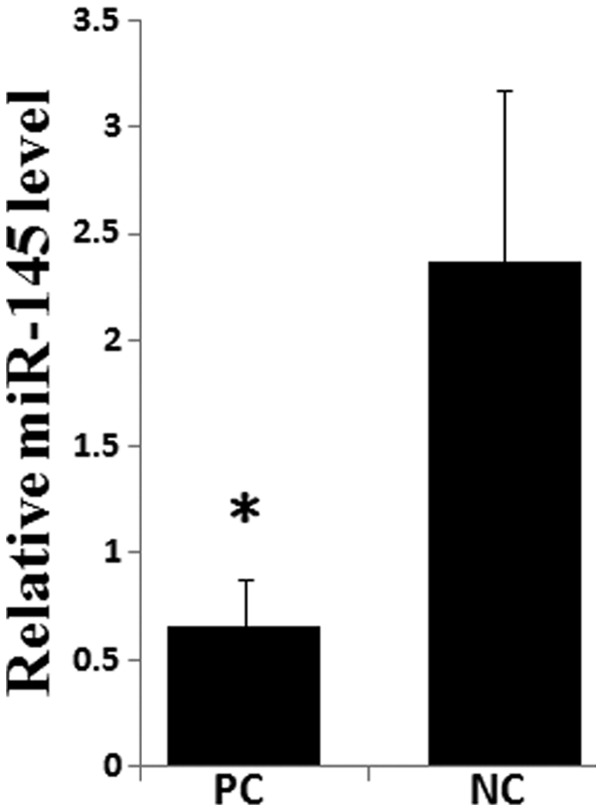
Expression of miR-145 in PC tissues and paired adjacent NC tissues *P<0.05, compared with control. miR, microRNA; PC, pancreatic cancer; NC, normal control.

miR-145 has been observed to be underexpressed in various types of cancer; however, its expression in PC has not been previously investigated, to the best of our knowledge. The expression of miR-145 was examined in different grades of PC using RT-qPCR. As shown in Fig. 1, the expression of miR-145 was significantly lower in PC tissues compared with paired adjacent normal pancreatic tissues (P<0.05). These data support the hypothesis that miR-145 may act as a tumor suppressor in PC.

### Cell proliferation is suppressed by re-expression of miR-145 in Panc-1 cells

**Figure 2:**
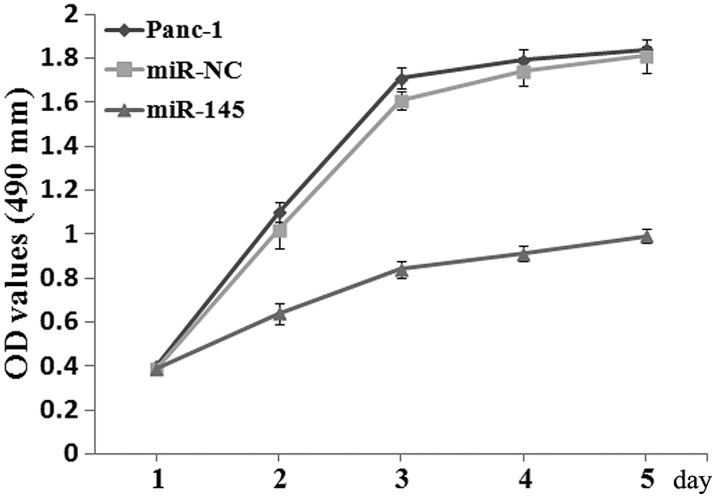
Effect of miR-145 restoration on Panc-1 cell growth. miR, microRNA; NC, normal control.

Initially, the effects of miR-145 on the proliferation of PC cells were investigated using an MTT assay. Panc-1 cells were transfected with 75 nM miR-145 mimics or miR-NC. The miR-145 mimics caused a 40-fold increase of the miR-145 expression in Panc-1 cells (data not shown). The MTT value of cells transfected with miR-145 mimics was significantly lower than that of cells transfected with miR-NC at 48 h post-transfection (P<0.05; Fig. 2).

### Cell invasion and migration are significantly decreased by re-expression of miR-145

**Figure 3:**
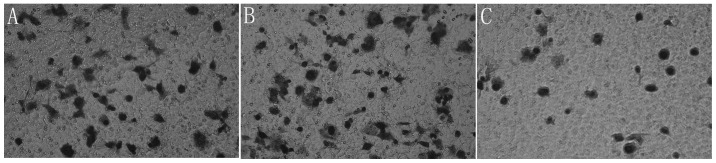
Effect of miR-145 restoration on cell invasion of Panc-1 cells via a Transwell chamber test. (A) Panc-1 cells, (B) Panc-1 cells + negative control miR and (C) Panc-1 cells + miR-145 (magnification, ×200). miR, microRNA.

**Figure 4:**
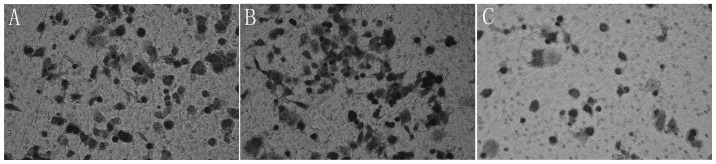
Effect of miR-145 restoration on cell migration of Panc-1 cells via a Transwell chamber test. (A) Panc-1 cells, (B) Panc-1 cells + negative control miR and (C) Panc-1 cells + miR-145 (magnification, ×200). miR, microRNA. miR, microRNA.

As PC is a malignant type of cancer with a potent capacity to invade locally and cause distant metastases, the effect of miR-145 restoration on Panc-1 cell invasion and migration was subsequently examined. Panc-1 cells were transfected with miR-145 mimics and then levels of cell invasion and migration were examined using a Transwell invasion and migration assay. The results revealed that the re-expression of miR-145 significantly decreased cell invasion and migration in Panc-1 cells (P<0.05; Figs. 3 and 4).

### miR-145 directly targets 3′UTR of NEDD9

**Figure 5:**
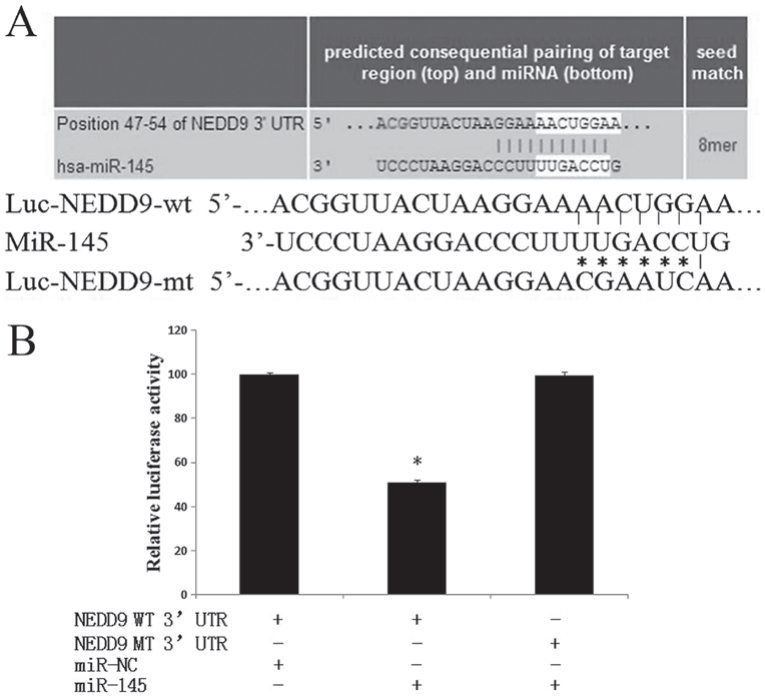
Luciferase assay results. (A) Wild-type 3′-UTR sequence or mutant 3′-UTR sequence of NEDD9 was cloned into luciferase plasmids (Luc-NEDD9-wt or Luc-NEDD9-mt, respectively). Binding sites are indicated by solid lines and mutant binding sites by ^*^. (B) Relative luciferase activity in groups transfected with different factors. ^*^P<0.05 vs. the NEDD9 WT + miR-NC and NEDD9 MT + miR-145 groups. NEDD9, neural precursor cell expressed, developmentally downregulated 9; UTR, untranslated region; Luc, luciferase; wt, wild-type; mt, mutant; miR, microRNA; NC, normal control.

To elucidate the molecular mechanism underlying miR-145 mediated regulation of proliferation, invasion and migration, *in silico* analysis was performed based on the computer-aided algorithms: PicTar, Targetscan, miRWalk and miRanda in conjunction with the miRGen Target program for predicted target genes. The most promising candidate was NEDD9, which was predicted by all of the applied algorithms. As shown in Fig. 5A, there is a putative 8-mer-binding site for miR-145 in the 3′UTR of the NEDD9 transcript [in addition to 7-mer sites, TargetScan predicts 8-mer sites defined as: An exact match to positions 2–8 of the mature miRNA (the seed+position 8) followed by an ‘A’]. In addition, it is a well-established metastasis- and migration-promoting gene, which is upregulated in several types of cancer, including PC. To identify whether miR-145 directly targets NEDD9, dual-luciferase reporter gene assays were performed. Luciferase reporter plasmids containing the wild-type 3′UTR (Luc-NEDD9-wt) or mutant 3′-UTR (Luc-NEDD9-mt) of NEDD9 were constructed to determine the targeted region (Fig. 5A). As shown in Fig. 5B, miR-145 significantly decreased the firefly luciferase activity in the reporter with wild type 3′UTR (P<0.05); however the activity of the mutant 3′UTR vector remained unaffected (P>0.05). These observations indicated that miR-145 directly targeted NEDD9 through interacting with the predicted binding site in its 3′UTR.

### miR-145 expression is inversely correlated with NEDD9 in PC

**Figure 6:**
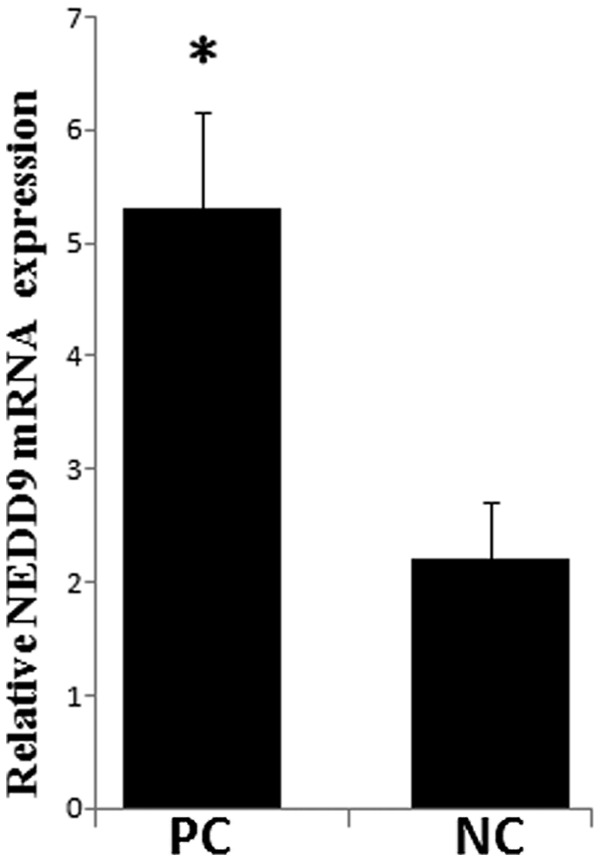
Expression of neural precursor cell expressed, developmentally downregulated 9; in PC tissue and paired adjacent NC tissues. ^*^P<0.05, compared with NC. PC, pancreatic cancer; NC, normal control.

**Figure 7:**
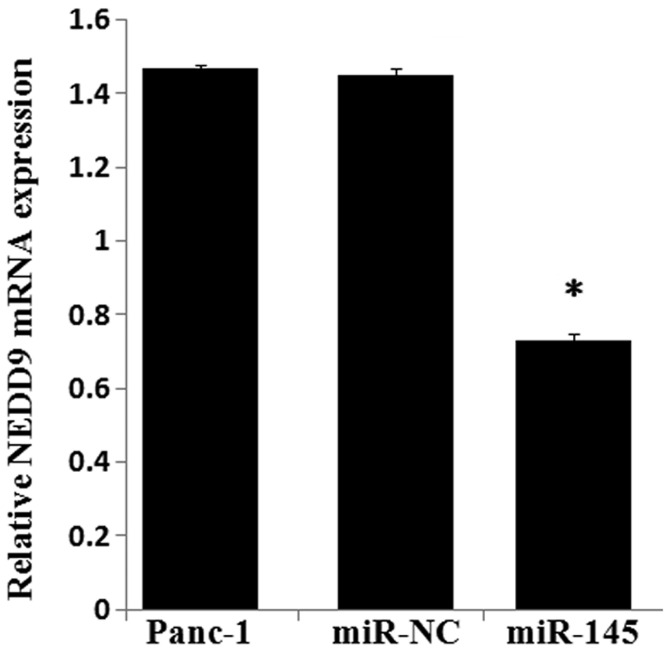
Effect of miR-145 restoration on NEDD9 mRNA expression in Panc-1 cells. ^*^P<0.05, compared with control. NEDD9, neural precursor cell expressed, developmentally downregulated 9; miR, microRNA; NC, normal control.

**Figure 8:**
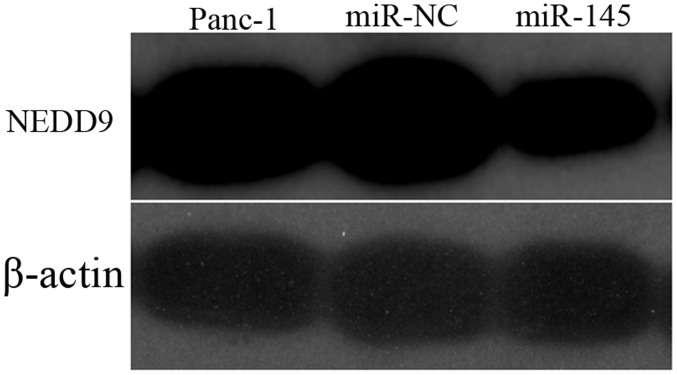
Effect of miR-145 restoration on NEDD9 protein expression in Panc-1 cells. NEDD9, neural precursor cell expressed, developmentally downregulated 9; miR, microRNA; NC, normal control.

The NEDD9 mRNA levels were measured in PC tissues and paired normal adjacent pancreatic tissues. As shown in Fig. 6, the average level of NEDD9 expression was significantly higher in PC tissues as compared with that of the NP tissues. A significant inverse correlation was observed between NEDD9 and miR-145 expression in PC tissues and adjacent noncancerous tissues. Transfection of Panc-1 cells with miR-145 mimics significantly decreased the expression of NEDD9 mRNA (Fig. 7) by 51% and the NEDD9 protein by 52.21% (Fig. 8) compared with levels in the control miR expressing cells (P<0.05).

## Discussion

miRNAs may negatively regulate gene expression by interacting with the specific target mRNA 3′UTR, which can result in gene silencing by either mRNA degradation or translation inhibition (17). miRNAs have been implicated in a broad range of biological processes, including cell proliferation, apoptosis, differentiation, metabolism, migration and invasion as each single miRNA may have more than one hundred targets and >30% of protein coding may be under the control of miRNAs (18). Mounting evidence indicates that deregulation of miRNA expression is often associated with a variety of disorders, including human cancer and numerous miRNAs have been observed to be differentially expressed in PC as compared with the normal pancreas (19,20). Since invasion and metastasis are the major causes of a poor prognosis in patients with PC, determining the complex role of specific miRNAs and their targets in the process of PC invasion and metastasis may provide novel insights for certain diagnoses and therapeutic consequences.

Aberrant expression of miR-145 has been observed in several types of cancer, including prostate, bladder, lung, breast and ovarian cancer. In addition, a close association between downregulated miR-145 and cancer invasion/metastasis has been observed (7–12). Microarray studies have identified that miR-145 was also downregulated in PC (13,14). However, the role of miR-145 in PC remains to be fully elucidated. In the present study, it was identified that miR-145 expression in PC tissues is significantly downregulated compared with that in adjacent normal pancreatic tissues, suggesting that miR-145 is a candidate tumor suppressor in the pathogenesis of PC. Downregulation or silencing of miR-145 may eliminate tumor suppression so as to contribute to tumorigenesis. Subsequently, the biological function of miR-145 in PC cells was examined and the results demonstrated that restoration of miR-145 in Panc-1 cells can significantly inhibit cell proliferation, invasion and migration, confirming previous studies suggesting a tumor-suppressive role for this miRNA. These results provided substantial evidence that miR-145 may be involved in the invasive and metastatic progression of PC. Therefore, approaches to introduce miR-145 into cancer cells may potentially be feasible in the clinical treatment of PC, particularly for patients with lower levels of miR-145 expression in their tumor tissues. However, the exact mechanism by which this treatment may aid therapy remains to be elucidated, largely due to the limited knowledge of miR-145 targets.

NEDD9, also termed HEF1 or Cas-L was initially identified by its developmentally regulated expression pattern in the early embryonic mouse brain. NEDD9 is a non-catalytic scaffolding protein, which is a member of the Crk-associated substrate (CAS) family (21). A series of studies have implicated the NEDD9 protein as a biomarker of invasive ability and an essential switch for pro-metastatic behavior in numerous types of cancer, including lung cancer, breast cancer and melanoma (22–24). The interaction of NEDD9 with FAK and Src leads to the tyrosine phosphorylation of NEDD9 to generate binding sites for effector proteins, including the Rac and the Cas-Crk complex, which then regulate and activate transcription pathways involved in metastasis and cancer progression (15). Recently, Speranza *et al* (25) identified that miR-145 is markedly downregulated whereas NEDD9 is significantly upregulated in glioblastoma specimens and corresponding glioblastoma-neurospheres (GB-NS) compared with normal brain and low-grade gliomas. The results suggested that miR-145 downregulates NEDD9, while NEDD9 down-regulates miR-145, forming a double-negative feedback loop in GB-NS (25). However, little is known about the function of NEDD9 in PC, and to the best of our knowledge, there are no previous studies investigating whether NEDD9 expression is regulated by specific miRNAs, such as miR-145, in PC.

In the present study, an important molecular association between miR-145 and NEDD9 was demonstrated. Firstly, the bioinformatics analysis indicated that NEDD9 may be one of the potential targets for miR-145. Subsequently, it was identified that NEDD9 is markedly upregulated in PC tissues, which is consistent with a previous study (26). More importantly, a significant negative correlation was observed between miR-145 and NEDD9 expression in PC tissues, and the expression of NEDD9 was decreased in Panc-1 cells accompanied by suppressed cell proliferation, invasion and migration when overexpressing miR-145, suggesting that miR-145 may be involved in the regulation of NEDD9 in PC. This hypothesis was further supported by the luciferase activity assay, in which the data demonstrated that miR-145 was able to directly target the 3′UTR of NEDD9 and the binding site was consistent with the seed region predicted by Targetscan. These data demonstrated that miR-145 may inhibit cell growth, invasion and migration of PC by targeting NEDD9.

In conclusion, data from the present study has demonstrated that miR-145 is downregulated in PC, restoration of miR-145 may inhibit the proliferation, migration and invasion capacity of PC cells by negatively regulating NEDD9 at the post-transcriptional level via directly binding to non-coding regions of NEDD9. The present data suggested that miR-145 may be useful as a novel potential therapeutic approach for the treatment of PC.
